# Efficacy of an herb and essential oil-based phytobiotic blend and its interaction with an antibiotic on intestinal health, growth performance, and carcass characteristics in growing pigs

**DOI:** 10.1093/jas/skaf370

**Published:** 2025-10-24

**Authors:** Jung Yeol Sung, Wanpuech Parnsen, Yesid Garavito-Duarte, Sung Woo Kim

**Affiliations:** Department of Animal Science, North Carolina State University, Raleigh, NC 27695; Department of Animal Science, North Carolina State University, Raleigh, NC 27695; Department of Animal Science, North Carolina State University, Raleigh, NC 27695; Department of Animal Science, North Carolina State University, Raleigh, NC 27695

**Keywords:** antibiotics, essential oils, herbs, phytobiotics, pig

## Abstract

Antibiotics were commonly added to pig diets to promote growth of pigs but are now restricted or no longer allowed worldwide due to concerns about antimicrobial resistance. However, because removing antibiotics from pig diets may compromise growth performance, researchers have actively explored alternative solutions. Phytobiotics, which are plant-derived compounds, are considered a potential alternative to antibiotics in pig diets. Therefore, the objective was to evaluate the effects of an herb and essential oil-based phytobiotic blend (HEP) on intestinal health, growth performance, and carcass characteristics in growing pigs fed diets with or without an antibiotic. A total of 96 pigs including 48 barrows and 48 gilts (initial weight = 41.5 ± 2.7 kg) were allotted to 4 dietary treatments based on a 2 × 2 factorial arrangements with the factors of antibiotic (0 or 0.05%) and HEP (0 or 0.05%). Pigs were fed the diets for 42 d and body weight and feed disappearance were recorded every 7 d. Blood samples were collected from each pig on d 37 to obtain plasma. On d 42, a pig with the median weight in each pen was euthanized to assess carcass characteristics and collect samples of tissue and mucosa from the duodenum and jejunum. The MIXED procedure of SAS was used to analyze data on intestinal health, growth performance, and carcass characteristics. The fixed effects were sex, antibiotic, HEP, and interaction between antibiotic and HEP, whereas initial body weight was a random effect. The supplementation of the HEP tended to decrease (*P *= 0.096) tumor necrosis factor-α in the jejunal mucosa. The supplementation of the HEP tended to increase villus height in the jejunum in pigs fed the diet without the antibiotic, whereas it had no effect on villus height in pigs fed the diet with the antibiotic (interaction; *P *= 0.061). The supplementation of the antibiotic increased (*P *< 0.05) average daily gain during the overall period. The supplementation of the HEP tended to decrease (*P *= 0.071) average daily feed intake, whereas it tended to increase (*P *= 0.087) gain:feed during the overall period. The supplementation of the antibiotic increased (*P *< 0.05) hot carcass weight, cold carcass weight, and loin color, and tended to decrease (*P *= 0.092) 48-h drip loss. In conclusion, supplementing antibiotic to the diets for growing pigs increased the weight gain resulting in increased hot and cold carcass weight, whereas supplementing the HEP increased the gain:feed of pigs without affecting the weight gain, which may be attributed to reduced inflammation in the jejunum. These results suggest that the HEP can be a potential alternative to the antibiotic, but the ways in which the antibiotic and HEP improve growth performance may differ.

## Teaser

The antibiotic increased weight gain, whereas the herb and essential oil-based phytobiotic increased feed efficiency of pigs.

## Introduction

Antibiotics have been used to treat both clinical and subclinical infections caused by pathogens including *Escherichia coli* and *Salmonella* (Wagner et al., 2008). In addition to treating infections, antibiotics also enhance growth performance in pigs, leading to their widespread inclusion in pig diets. Currently, antibiotics are now restricted or no longer allowed in pig diets worldwide due to concerns about antimicrobial resistance (Zhu et al., 2013; FDA, 2023; Sung et al., 2025). However, removing antibiotics in pig diets may increase the intestinal inflammation and metabolic cost associated with activating immune responses during sub-clinical or clinical infections, potentially compromising growth performance (Duarte et al., 2023). Potential alternatives to antibiotics include probiotics (Casas et al., 2020; Duarte et al., 2020; Sun et al., 2021), prebiotics (Houdijk et al., 1998; Li et al., 2021), postbiotics (Xu et al., 2022; Duarte and Kim, 2024), and phytobiotics (Kommera et al., 2006; Hanczakowska and Swiatkiewicz, 2012; Garavito-Duarte et al., 2025a).

Phytobiotics are defined as plant-derived compounds incorporated into diets to improve the productivity of livestock (Windisch et al., 2008). Phytobiotics consist of various components, with herbs and essential oils being common components of phytobiotics (Piao et al., 2006; Garavito-Duarte et al., 2025b). The supplementation of phytobiotics to pig diets potentially improves growth performance by positively modulating intestinal microbiota (Moita et al., 2021; Duarte and Kim, 2022a; Garavito-Duarte et al., 2025c), reducing oxidative stress (Kaschubek et al., 2018; Peng et al., 2022), and preventing damage to villi in the small intestine (Zeng et al., 2015; Xu et al., 2018). Most research on phytobiotics has focused on alleviating post-weaning stress in nursery pigs (Garavito-Duarte et al., 2025b). However, phytobiotics may also benefit growing-finishing pigs by reducing oxidative damage in muscle tissue and enhancing carcass characteristics, such as water-holding capacity and color (Janz et al., 2007; Zhou et al., 2013; Cheng et al., 2017). Depending on major components, beneficial effects of phytobiotics could be based on distinctly different mechanisms. Herb-based phytobiotics can limit the proliferation of potentially pathogenic bacteria in the intestines, whereas essential oil-based phytobiotics can promote the growth of beneficial bacteria, both contributing to the positive modulation of intestinal microbiota in pigs (Namkung et al., 2004; Garavito-Duarte et al., 2025c). This positive modulation of intestinal microbiota by both types of phytobiotics may contribute to enhanced intestinal health and improved growth performance of pigs (Kommera et al., 2006; Li et al., 2012; Zhao et al., 2017). The difference in their mechanisms underlying the modulation of intestinal microbiota is attributable to their main bioactive compounds (Cowan, 1999): alkaloids and phenolic compounds in herb-based phytobiotics, and aliphatic hydrocarbons (e.g., thymol and eugenol), aromatic aldehydes (e.g., cinnamaldehyde), and terpenes in essential oil-based phytobiotics (Gill and Holley, 2006; Kantas et al., 2015; Garavito-Duarte et al., 2025c). Given the distinct mechanisms resulting from their different combinations of bioactive compounds with functional properties, a blend of herb-based and essential oil-based phytobiotics may provide complementary benefits for pigs.

Therefore, this study aimed to test the hypothesis that an herb and essential oil-based phytobiotic blend (HEP), as an alternative to antibiotic use, improves intestinal health, growth performance, and carcass characteristics in growing pigs. This hypothesis is based on the premise that the HEP is an effective alternative to antibiotics in pigs due to the diverse bioactive compounds of herb and essential oil-based phytobiotics. To test this hypothesis, the objective was to evaluate the effects of the HEP on intestinal health, growth performance, and carcass characteristics in growing pigs fed diets with or without an antibiotic.

## Materials and Methods

The protocol of this study was reviewed and approved by Animal Care and Use Committee (REG 10.10.01) of North Carolina State University (Raleigh, NC, USA).

### Animals, experimental design, and experimental diets

This study was conducted at the swine research facility of North Carolina State University (Clayton, NC, USA). A total of 96 pigs including 48 barrows and 48 gilts (PIC 337 × Camborough 22; initial weight = 41.5 ± 2.7 kg) were allotted to 4 dietary treatments (Table 1) based on a 2 × 2 factorial arrangements with 0 or 0.05% of antibiotic (Tylan40, Elanco, Indianapolis, IN, USA) and 0 or 0.05% of the HEP (AV/AGP/10, Ayurvet, Baddi, India). The primary components of the HEP used in the study are *Eucalyptus globulus*, *Cichorium intybus*, and *Trigonella foenum graecum*, with addition of *Allium sativum, Zingiber officinale*, and *Mentha piperita*. The pigs were allotted to the treatments based on a randomized complete block design with initial body weight (heaviest, heavy intermediate, light intermediate, and lightest) and sex (barrow and gilt) as blocks. The diets were formulated to meet or exceed nutrient requirements provided by NRC (2012). Each treatment included eight pens (four barrow and four gilt pens) with three pigs per pen and each pen was equipped with a self-feeder and nipple. Each pig was ear-tagged for individual identification. Each pen had a solid concrete floor (2 m × 2 m). Pigs had unlimited access to mashed form of feed and water. Pigs were fed the diets for 42 d, and body weight and feed disappearance were recorded every 7 d. All pigs exhibited no clinical signs of illness and maintained normal health status.

**Table 1.** Composition of diets (as-fed basis)

Antibiotic^1^, %	0		0.05	
**Herb and essential oil-based phytobiotic blend^2^, %**	0	0.05	0	0.05
**Feedstuff, %**				
** Corn**	69.45	69.40	69.40	69.35
** Soybean meal**	17.20	17.20	17.20	17.20
** Corn distillers dried grains with solubles**	10.00	10.00	10.00	10.00
** L-lysine HCl**	0.27	0.27	0.27	0.27
** L-threonine**	0.03	0.03	0.03	0.03
** Dicalcium phosphate**	0.70	0.70	0.70	0.70
** Limestone**	0.95	0.95	0.95	0.95
** Poultry fat**	1.00	1.00	1.00	1.00
** Salt**	0.22	0.22	0.22	0.22
** Vitamin premix^3^**	0.03	0.03	0.03	0.03
** Mineral premix^4^**	0.15	0.15	0.15	0.15
** Antibiotic**	–	–	0.05	0.05
** Herb and essential oil-based ­phytobiotic blend**	–	0.05	–	0.05
**Calculated composition^5^**				
** ME, kcal/kg**	3,361	3,359	3,359	3,357
** SID lysine, %**	0.85	0.85	0.85	0.85
** SID methionine + cysteine, %**	0.49	0.49	0.49	0.49
** SID tryptophan, %**	0.15	0.15	0.15	0.15
** SID threonine, %**	0.52	0.52	0.52	0.52
** Calcium, %**	0.59	0.59	0.59	0.59
** STTD phosphorus, %**	0.27	0.27	0.27	0.27

1 Tylan40 (Elanco, Indianapolis, IN, USA).

2 AV/AGP/10 (Ayurvet, Baddi, India).

3 The vitamin premix provided the following per kilogram of complete diet: 6,614 IU of vitamin A; 992 IU of vitamin D_3_; 19.8 IU of vitamin E; 2.64 mg of vitamin K; 0.03 mg of vitamin B_12_; 4.63 mg of riboflavin; 18.52 mg of pantothenic acid; 24.96 mg of niacin; 0.07 mg of biotin.

4 The trace mineral premix provided the following per kilogram of complete diet: 4.0 mg of Mn as manganous oxide; 165 mg of Fe as ferrous sulfate; 165 mg of Zn as zinc sulfate; 16.5 mg of Cu as copper sulfate; 0.30 mg of I as ethylenediamine dihydroiodide; and 0.30 mg of Se as sodium selenite.

5 ME, metabolizable energy; SID, standardized ileal digestible; STTD, standardized total tract digestible.

### Sampling

On d 37, blood samples were collected from the jugular vein of each pig using Monovette tubes (Sarstedt, Newton, NC, USA) containing ethylenediaminetetraacetic acid to obtain plasma. Subsequently, the collected blood samples were centrifuged at 3,000×*g* for 15 min at 4°C and stored at −80°C. On d 42, a pig with the median body weight in each pen was euthanized using percussive stunning and exsanguination. Subsequently, duodenal tissue (from 30 cm after the pyloric duodenal junction) and jejunal tissue (from 3 m after the pyloric duodenal junction) were collected (a section of 20 cm) and rinsed with saline to remove any residual digesta. The mucosa from the first 15 cm of tissues was scraped using a glass slide, transferred into 2-mL tubes, frozen in liquid nitrogen, and stored at −80°C for further analysis. The remaining 5 cm of the jejunum was placed in a 50 mL-tube containing 10% buffered formaldehyde solution for further evaluation of intestinal morphology.

### Immune response

A homogenizer (Tissuemiser, Thermo Fisher Scientific Inc., Waltham, MA, USA) was used to homogenize one gram of duodenal and jejunal mucosa with 1 mL of phosphate-buffered saline for tumor necrosis factor-α (TNF-α) analysis. The homogenized sample was centrifuged at 14,000×*g* for 30 min at 4 °C, and the resulting supernatant was collected. The total protein content in the supernatant was determined using the Pierce BCA Protein Assay kit (23225# Thermo Fisher Scientific Inc., Waltham, MA, USA). The standard curve was used to calculate the total protein concentration (mg/mL), which was then used to normalize the concentration of TNF-α in the plasma, duodenal mucosa, and jejunal mucosa. Plasma and mucosa samples were diluted to fall within the working range of 20 to 2,000 μg/mL, and absorbance was measured at 562 nm using a microplate reader (Synergy^TM^ HT, Biotek, Charlotte, VT, USA). The concentration of TNF-α in the plasma and duodenal and jejunal mucosa was quantified using ELISA kits (R&D Systems, Minneapolis, MN, USA) based on the method described by the manufacturer’s instruction and the absorbance was measured at 450 nm. The concentration of TNF-α in plasma was expressed as pg/mL of protein, and in the duodenal and jejunal mucosa as pg/mg of protein.

### Intestinal morphology in the jejunum

After fixation in 10% buffered formaldehyde for 48 h, the collected jejunal tissues, approximately 2 mm in length, were cut transversely, placed in a cassette, and immersed in a 70% ethanol solution. The samples were then sent to the Histology Laboratory at North Carolina State University’s College of Veterinary Medicine (Raleigh, NC, USA) for dehydration, embedding, and staining. Villus height and crypt depth were measured using an Olympus CX31 microscope (Lumenera Corporation, Ottawa, Canada) with Infinity 2-2 digital CCD software at 40× magnification, following the method outlined by Baker et al. (2025). For each pig, 10 intact villi and their associated crypts were measured. The height of the villus was measured from the top of the villus to the junction of villus and crypt depth was measured from the junction of villus and crypt to the bottom of the crypt. The villus height to crypt depth ratio was calculated by dividing measured villus height by crypt depth.

### Carcass characteristics

After euthanizing pigs, hot carcass weight was recorded. Dressing percentage was determined using the following calculation:


Dressing percentage%=hot carcass weight kg  ÷final live weightkg×100

Carcasses were chilled for 24 h at 4 °C, then cold carcass weight was measured. Dorsal subcutaneous adipose tissue thickness was measured in millimeters at the first, tenth, and last ribs using a ruler. Boneless loin was removed from the right side of each carcass, immediately identified, and weighed. The loin was collected from tenth rib location, and a 250 g from the collected loin was used for marbling evaluation, loin eye area measurement, and drip loss determination. Color of loin was assessed using a color panel scale ranging from 1 to 6 (very pale to very dark) based on NPPC (1991). Marbling of loin was scored from 1 (devoid) to 5 (moderately abundant or greater) based on NPPC (1999). The surface of each loin eye section was traced on transparency paper, and loin eye area was measured using grid paper in square centimeters (National Pork Board, 2000). A 3-cm slice from the cranial end of each loin eye section was weighed (W1), placed in a sealed polyethylene plastic bag, and stored at 4°C. After 48 h, each slice was weighed (W2) and 48 h-drip loss was determined using the following calculation:


$$\begin{array}{c} 48\;h-\mathrm{drip}\;\mathrm{loss}\;\left(\%\right)\;=\;\left(W1-W2\right)\;/\;W1\;\times \;100 \end{array}$$

### Statistical analysis

The MIXED procedure of SAS (SAS Institute Inc., Cary, NC, USA) was used to analyze data on intestinal health, growth performance, and carcass characteristics. Sex, antibiotic, HEP, and interaction between antibiotic and HEP were the fixed effects, whereas initial body weight (heaviest, heavy intermediate, light intermediate, and lightest) was a random effect. When there was an interaction, differences between least squares means were tested using the PDIFF option with Tukey’s adjustment. Plasma samples were collected from each pig, and the average TNF-α concentration in the plasma per pen was used for statistical analysis. A pen was the experimental unit for TNF-α in plasma and growth performance, whereas pig was the experimental unit for carcass characteristics, TNF-α in the duodenal and jejunal mucosa, and intestinal morphology. Significance and tendency were determined at *P *< 0.05 and 0.05 ≤ *P *< 0.10, respectively.

## Results

The supplementation of the HEP tended to decrease (*P *= 0.096) TNF-α in the jejunal mucosa but not in the plasma and duodenal mucosa (Table 2). The supplementation of the HEP tended to increase villus height in the jejunum in pigs fed the diet without the antibiotic, whereas it had no effect on villus height in pigs fed the diet with the antibiotic (interaction; *P *= 0.061; Table 3). The supplementation of the antibiotic tended to increase (*P *= 0.082) crypt depth in the jejunum. The supplementation of the antibiotics or HEP did not affect villus height to crypt depth in the jejunum. The supplementation of the antibiotic increased (*P *< 0.05) average daily gain during the overall period, whereas the HEP had no effect (Table 4). The supplementation of the HEP tended to reduce average daily gain (*P *= 0.083) during d 21 to 28. The supplementation of the HEP reduced average daily feed intake in pigs fed the diet without the antibiotic, whereas it had no effect on average daily feed intake in pigs fed the diet with the antibiotic from d 28 to 35 (interaction; *P *< 0.05). The supplementation of the HEP tended to reduce average daily feed intake during d 21 to 28 (*P *= 0.079) and the overall period (*P *= 0.071). The supplementation of the HEP tended to increase gain:feed in pigs fed the diet without the antibiotic, whereas it had no effect on gain:feed in pigs fed the diet with the antibiotic from d 0 to 7 (interaction; *P *= 0.058). The supplementation of the HEP increased (*P *< 0.05) gain:feed from d 28 to 35 and d 35 to 42. The supplementation of the HEP tended to increase (*P *= 0.087) gain:feed during the overall period. The supplementation of the antibiotic increased (*P *< 0.05) hot carcass weight, cold carcass weight, and loin color, whereas tended to decrease (*P *= 0.092) 48-h drip loss (Table 5). The supplementation of the HEP did not affect carcass characteristics.

**Table 2.** Concentration of tumor necrosis factor-alpha (TNF-α) in plasma and intestinal mucosa of pigs fed diets supplemented with antibiotic or herb and essential oil-based phytobiotic blend^1^

Antibiotic^2^, %	0		0.05			*P*-value^4^		
Herb and essential oil-based phytobiotic blend^3^, %	0	0.05	0	0.05	SEM	Antibiotic	Phytobiotic	Interaction
**Plasma, pg/mL**	47.3	43.0	43.2	43.0	2.6	0.440	0.408	0.451
**Duodenum, pg/mg of protein**	5.3	5.3	4.7	4.9	0.6	0.395	0.972	0.868
**Jejunum, pg/mg of protein**	3.4	2.7	3.1	2.8	0.3	0.815	0.096	0.613

1 Each least squares mean represent 8 observations.

2 Tylan40 (Elanco, Indianapolis, IN, USA).

3 AV/AGP/10 (Ayurvet, Baddi, India).

4 Phytobiotic, herb and essential oil-based phytobiotic blend; Interaction, interaction between antibiotic and herb and essential oil-based phytobiotic blend.

**Table 3.** Jejunal morphology of pigs fed diets supplemented with antibiotic or herb and essential oil-based phytobiotic blend^1^

Antibiotic^2^, %	0		0.05			*P*-value^4^		
**Herb and essential oil-based phytobiotic blend** ^3^ **, %**	0	0.05	0	0.05	SEM	Antibiotic	Phytobiotic	Interaction
**Villus height, μm**	365^A^	372^B^	379^C^	377^C^	4	0.006	0.189	0.061
**Crypt depth, μm**	247	251	253	254	2	0.082	0.265	0.567
**Villus height to crypt depth ratio**	1.47	1.48	1.50	1.49	0.01	0.290	0.878	0.444

A–C Least squares means without a common uppercase superscript tend to be different (0.05 ≤ *P *< 0.10).

1 Each least squares mean represent 8 observations.

2 Tylan40 (Elanco, Indianapolis, IN, USA).

3 AV/AGP/10 (Ayurvet, Baddi, India).

4 Phytobiotic, herb and essential oil-based phytobiotic blend; Interaction, interaction between antibiotic and herb and essential oil-based phytobiotic blend.

**Table 4.** Growth performance of pigs fed diets supplemented with antibiotic or herb and essential oil-based phytobiotic blend^1^

Antibiotic^2^, %	0		0.05			*P*-value^4^		
**Herb and essential oil-based phytobiotic blend^3^, %**	0	0.05	0	0.05	SEM	Antibiotic	Phytobiotic	Interaction
**Body weight, kg**								
** d 0**	41.5	41.5	41.3	41.5	3.5	0.936	0.864	0.854
** d 7**	47.2	48.0	48.1	47.6	3.7	0.671	0.871	0.269
** d 14**	54.3	54.9	55.2	54.9	3.8	0.609	0.860	0.550
** d 21**	61.6	62.9	63.6	62.9	4.2	0.263	0.698	0.235
** d 28**	69.8	70.3	71.9	71.0	4.3	0.077	0.776	0.384
** d 35**	78.1	78.8	80.3	80.0	4.4	0.067	0.851	0.622
** d 42**	88.0	89.0	90.5	90.3	4.7	0.064	0.686	0.543
**Average daily gain, kg/d**								
** d 0 to 7**	0.82^a^	0.93^ab^	0.97^b^	0.86^a^	0.05	0.417	0.961	0.044
** d 7 to 14**	1.01	1.00	1.01	1.04	0.05	0.633	0.887	0.615
** d 14 to 21**	1.03	1.15	1.20	1.15	0.08	0.131	0.602	0.131
** d 21 to 28**	1.18	1.05	1.19	1.16	0.05	0.162	0.083	0.269
** d 28 to 35**	1.19	1.21	1.20	1.29	0.04	0.293	0.142	0.357
** d 35 to 42**	1.97	2.05	2.05	2.06	0.09	0.483	0.446	0.589
** d 0 to 42 (overall)**	1.16	1.19	1.23	1.22	0.04	0.021	0.761	0.365
**Average daily feed intake, kg/d**								
** d 0 to 7**	1.97	1.97	2.08	1.89	0.12	0.853	0.268	0.287
** d 7 to 14**	2.44	2.43	2.40	2.45	0.14	0.900	0.802	0.695
** d 14 to 21**	2.66	2.59	2.63	2.56	0.16	0.643	0.236	0.916
** d 21 to 28**	3.01	2.89	3.04	2.98	0.16	0.230	0.079	0.536
** d 28 to 35**	2.81^b^	2.65^a^	2.81^b^	2.85^b^	0.12	0.040	0.214	0.045
** d 35 to 42**	3.28	3.16	3.34	3.29	0.18	0.173	0.267	0.636
** d 0 to 42 (overall)**	2.83	2.74	2.85	2.80	0.14	0.233	0.071	0.629
**Gain to feed ratio**								
** d 0 to 7**	0.42^A^	0.47^B^	0.47^B^	0.45^AB^	0.02	0.321	0.391	0.058
** d 7 to 14**	0.42	0.41	0.43	0.43	0.02	0.399	0.979	0.865
** d 14 to 21**	0.40	0.45	0.46	0.45	0.02	0.159	0.375	0.210
** d 21 to 28**	0.39	0.37	0.39	0.39	0.02	0.407	0.308	0.355
** d 28 to 35**	0.43	0.46	0.43	0.46	0.02	0.825	0.047	0.691
** d 35 to 42**	0.61	0.66	0.62	0.63	0.02	0.618	0.046	0.285
** d 0 to 42 (overall)**	0.41	0.44	0.43	0.44	0.01	0.199	0.087	0.255

a-b Least squares means within a row without a common superscript differ (*P *< 0.05).

A-B Least squares means without a common uppercase superscript tend to be different (0.05 ≤ *P *< 0.10).

1 Each least squares mean represent 8 observations.

2 Tylan40 (Elanco, Indianapolis, IN, USA).

3 AV/AGP/10 (Ayurvet, Baddi, India).

4 Phytobiotic, herb and essential oil-based phytobiotic blend; Interaction, interaction between antibiotic and herb and essential oil-based phytobiotic blend.

**Table 5.** Carcass characteristics of pigs fed diets supplemented with antibiotic or herb and essential oil-based phytobiotic blend^1^

Antibiotic^2^, %	0		0.05			*P*-value^4^		
Herb and essential oil-based phytobiotic blend^3^, %	0	0.05	0	0.05	SEM	Antibiotic	Phytobiotic	Interaction
**Live weight, kg**	87.9	87.7	88.8	89.7	4.7	0.299	0.815	0.707
**Hot carcass, kg**	62.1	61.9	64.3	63.2	3.4	0.041	0.431	0.606
**Cold carcass, kg**	60.0	59.9	62.3	61.2	3.3	0.038	0.436	0.546
**Dressing percentage, %**	70.7	70.6	72.5	70.4	0.7	0.231	0.101	0.135
**Back fat thickness, mm**								
** 1^st^ Rib**	27.6	28.8	27.6	26.0	2.2	0.501	0.902	0.501
** 10^th^ Rib**	18.9	18.4	18.3	19.0	1.8	0.950	0.924	0.633
** Last rib**	25.5	22.9	23.8	23.1	1.7	0.627	0.296	0.518
**Loin quality**								
** Loin weight, kg**	2.0	2.0	2.0	2.0	0.1	0.359	0.685	0.946
** Loin eye area, cm^2^**	48.8	48.6	49.4	49.8	3.0	0.283	0.887	0.715
** Loin color**	2.1	2.1	2.4	2.5	0.2	0.046	0.678	0.686
** Marbling score**	2.1	2.5	2.1	2.1	0.3	0.470	0.470	0.470
** 48-h drip loss, %**	3.9	3.8	3.6	3.5	0.2	0.092	0.404	0.914

1 Each least squares mean represent 8 observations.

2 Tylan40 (Elanco, Indianapolis, IN, USA).

3 AV/AGP/10 (Ayurvet, Baddi, India).

4 Phytobiotic, herb and essential oil-based phytobiotic blend; Interaction, interaction between antibiotic and herb and essential oil-based phytobiotic blend.

## Discussion

Phytobiotics have the potential to replace antibiotics in pig diets (Kommera et al., 2006; Kim et al., 2008; Windisch et al., 2008). The growth-promoting effects of antibiotics and phytobiotics are potentially attributed to improved intestinal health, potentially resulting from their antimicrobial effects. However, the antimicrobial effects of the HEP may differ in mechanisms from those of antibiotics. Specifically, the antibiotic used in this study (Tylan40 classified as a macrolide antibiotic) suppresses bacterial growth by inhibiting protein synthesis (Hansen et al., 2002; Sung et al., 2025). In contrast, the antimicrobial mechanisms of the HEP used in this study are not limited to inhibiting bacterial growth but also include other mechanisms. The HEP used in this study consists of herbs (*Cichorium intybus*, *Trigonella foenum graecum*, *Allium sativum*, and *Zingiber officinale*) mainly composed of alkaloids, flavonoids, and phenolic compounds (Shen et al., 2003; Hossain et al., 2018; Perovic et al., 2021; Baies et al., 2024) and essential oils (*Eucalyptus globulus* and *Mentha piperita*) mainly composed of terpenes (Shao et al., 2020; Hejna et al., 2021). The key components in herb-based phytobiotics (alkaloids, flavonoids, and phenolic compounds) can intercalate with bacterial DNA, causing it to unwind and elongate, inhibit membrane-bound enzymes (e.g., glucan synthase) in bacteria, and bind to bacterial DNA or RNA, altering their structure and disrupting transcription and translation processes (Mason and Wasserman, 1987; Phillipson and O′Neill, 1987; Du et al., 2020). In contrast, terpenes in essential oil-based phytobiotics target the lipid components of bacterial membranes in both gram-negative and gram-positive bacteria, altering membrane permeability and leading to the leakage of cellular contents (Trombetta et al., 2005; El Fannassi et al., 2023). In addition, certain terpenes commonly found in essential oils, such as eucalyptol and menthol, can activate transient receptor potential melastatin eight receptors, which in turn regulate the Nrf2/HO-1 and NF-κB pathways, contributing to anti-inflammatory effects (Caceres et al., 2017; Ghasemi-Pirbaluti et al., 2017; Gao et al., 2025). Thus, differences in antimicrobial and immunomodulatory mechanisms between the antibiotic and the HEP used in the study may lead to distinct effects on intestinal health and pig growth.

Tumor necrosis factor-α is one of the major pro-inflammatory cytokines that induce inflammation in the intestinal tract related to villus destruction in pigs (Duarte and Kim, 2022b). The concentration of TNF-α in the jejunal mucosa of growing pigs fed diets without the antibiotic and the HEP (3.4 pg/mg of protein) in this study was greater than the average values (1.0 pg/mg of protein) reported in recent studies for nursery pigs (Deng et al., 2023; Duarte et al., 2023; Baker et al., 2024; Duarte et al., 2024a; Duarte and Kim, 2024; Baker et al., 2025; Cheng et al., 2025; Choi et al., 2025). The increased TNF-α in the jejunal mucosa is a potential indicator of elevated intestinal inflammation because TNF-α is one of the major pro-inflammatory cytokines released early during immune activation (Lonnemann et al., 1989; Zelnickova et al., 2008). When TNF-α is released, it serves as a key initiator of the inflammatory cascade by promoting vasodilation and edema, enhancing leukocyte adhesion through upregulation of adhesion molecules, influencing coagulation, and inducing oxidative stress (Zelová and Hošek, 2013). The reason for the greater TNF-α concentration in the jejunal mucosa of growing pigs in this study compared with nursery pigs in the previous studies remains unclear. It is speculated that the activation of macrophages and other immune cells is more pronounced as pigs grow, leading to greater TNF-α concentrations in the jejunal mucosa of growing pigs compared with nursery pigs (Zelnickova et al., 2008; Balasch et al., 2019). In this study, the tendency decrease of TNF-α in the jejunal mucosa by the HEP may be partially attributed to the modulation of intestinal microbiota and the inhibition of inflammatory pathways as previously shown by Duarte and Kim (2022a). Previous studies reported that the supplementation of herb or essential oil-based phytobiotics containing components present in the HEP such as *Allium sativum*, *Cichorium intybus*, *Trigonella foenum graecum, Mentha piperita*, and *Eucalyptus globulus* reduced the relative abundance of potentially pathogenic bacteria (e.g., *Escherichia coli*), whereas increased the relative abundance of potentially beneficial bacteria (e.g., *Lactobacillus*, *Bifidobacteria*) in the intestinal tract of pigs (Ruzauskas et al., 2020; Garavito-Duarte et al., 2025c). This positive modulation of intestinal microbiota by the HEP might have suppressed the expression of pattern recognition receptors and transcription factors which trigger immune responses and then activate pro-inflammatory cascade, leading to the reduced TNF-α in the jejunal mucosa (Duarte et al., 2024a). Subsequently, the reduced TNF-α or bioactive compounds in the HEP (e.g., flavonoids, phenolic compounds, and terpenes) may suppress intestinal inflammation by inhibiting the NF-κB pathway (Dou et al., 2013) and mitogen-activated protein kinase pathway through targeting extracellular signal-regulated kinase and c-Jun N-terminal kinase signaling pathways (Rodríguez-Ramiro et al., 2013; Ran et al., 2018; Wang et al., 2024). In contrast, tylosin did not affect TNF-α concentrations in the plasma and mucosa of pigs in this study, consistent with previous studies (Wang et al., 2018; Liao et al., 2023). However, other types of antibiotics such as bacitracin (Duarte et al., 2024b) and carbadox reduced the concentrations of TNF-α, interleukin-1β, and interleukin-8 in the intestine of pigs (Hung et al., 2020; Choi et al., 2025), indicating that the impacts of antibiotics on the production of pro-inflammatory cytokines vary depending on the mechanisms of each antibiotic (Sung et al., 2025). Additionally, the immunomodulatory effects of tylosin have been reported to be more pronounced in challenge situations with lipopolysaccharide or pathogens such as *Actinobacillus pleuropneumoniae* and *Pasteurella multocida* (Cao et al., 2006; Lee et al., 2024). This might explain why tylosin showed limited effects under the non-challenged conditions of this study.

The supplementation of the HEP tended to increase villus height in the jejunum in pigs fed the diet without the antibiotic, which is partially attributed to a decrease in TNF-α in the jejunal mucosa because pro-inflammatory cytokines induce intestinal morphologic changes (Sun et al., 2021). Specifically, pro-inflammatory cytokines can potentially increase cell death through apoptosis and induce oxidative damage to intestinal cells, which may reduce villus height in the jejunum (Schmitz et al., 1999; Duarte et al., 2020). Some bioactive compounds in phytobiotics, such as flavonoids and tannins, possess anti-oxidative properties that may attenuate inflammatory response and help preserve epithelial integrity (Pei et al., 2020; Tong et al., 2021). Moreover, the supplementation with the bioactive compounds in the HEP used in this study (*Eucalyptus globulus*, *Cichorium intybus*, *Trigonella foenum-graecum*, *Allium sativum*, *Zingiber officinale*, and *Mentha piperita*) increased villus height, enhanced the expression of tight junction proteins in the small intestine, and lowered intestinal pH by increasing lactic acid concentrations (Zentek et al., 2013; Rabelo-Ruiz et al., 2021; Chen et al., 2025). Additionally, it also improved antioxidant capacity, reduced oxidative stress and inflammation both locally and systemically, and modulated the intestinal microbiota by decreasing the relative abundance of pathogenic bacteria whereas promoting beneficial bacteria in both the digesta and jejunal mucosa (Liu et al., 2012; Herve et al., 2023; Garavito-Duarte et al., 2025c). However, the supplementation of the HEP did not affect villus height in the jejunum when pigs were fed the diet with the antibiotic, indicating that the antibiotic might have masked the positive effects of the HEP on jejunal villus height. This is partly attributed to the antimicrobial and anti-oxidative properties of the antibiotic, as supported by studies showing increased villus height and reduced oxidative stress (Xu et al., 2022; Choi et al., 2025). However, the supplementation of the antibiotic tended to increase crypt depth of the jejunum (indicating an increased cellular turnover and renewal requirement for the villi), suggesting that antibiotics may not always benefit intestinal health of pigs (May et al., 2012).

In this study, growth response of pigs was different between dietary supplementation of the antibiotic and the HEP. The supplementation of the antibiotic increased average daily gain regardless of the HEP during the overall period in this study, which is consistent with previous results (Gormley et al., 2024; Kim et al., 2025). The improvement in average daily gain from the antibiotic is partly due to the suppression of subclinical immune responses and the overall intestinal microbiota, allowing energy and nutrients to support growth rather than being used to activate immune responses and microbial activity (Cromwell, 2002). In addition, the supplementation of the antibiotic might have contributed to increased average daily gain by increasing ileal digestibility of nitrogen and amino acids (Roth and Kirchgessner, 1993). The improvement in digestibility from the antibiotic might result from a thinner mucosal wall, reduced bacterial protein synthesis, and decreased inactivation of pancreatic enzymes by microbiota in the small intestine (Corring et al., 1981; Eidelsburger et al., 1992). In contrast to the antibiotic, the supplementation of the HEP tended to reduce average daily feed intake of pigs without affecting average daily gain, resulting in improved gain:feed. The tendency on the reduction in average daily feed intake may be attributed to the distinct odor and flavor of the HEP (Kommera et al., 2006; Garavito-Duarte et al., 2025b). To address the common drawback of feed intake depression associated with phytobiotics, a titration study is warranted to determine the optimal concentration that enhances intestinal health without compromising intake (Windisch and Kroismayr, 2006). However, the reduction in average daily feed intake caused by the HEP was not observed from d 28 to 35 when the antibiotic was included, suggesting that antibiotics may promote average daily feed intake (Bosi et al., 2011). The HEP improved gain:feed without compromising weight gain during the overall period, likely by reducing intestinal inflammation (TNF-α). The reduced TNF-α in the jejunal mucosa may have lowered the metabolic cost of inflammation and improved efficiency of nutrient utilization, thereby offsetting the potential negative impact of reduced feed intake on average daily gain (Mercier et al., 2002). Additionally, the reduction in TNF-α in the jejunal mucosa by the HEP may have contributed to the increased jejunal villus height in pigs fed diets without antibiotics, which could have also contributed to the increased gain:feed by improving the capacity of nutrient absorption. However, it should be noted that the HEP was effective in reducing the concentration of only one specific inflammatory cytokine in the jejunal mucosa. In accordance with the improved average daily gain, the supplementation of the antibiotic increased hot and cold carcass weights in this study. However, the supplementation of either the antibiotic or the HEP did not affect lean-to-fat ratio in pigs, as evidenced by the lack of change in dressing percentage, back fat thickness, loin weight, and marbling score. The supplementation of the antibiotic increased loin color, resulting in a darker appearance that is more appealing to consumers (Moody et al., 1980; Richardson et al., 2018). The supplementation of the antibiotic may have promoted a shift toward a higher proportion of type I muscle fibers and a reduction in type II fibers, resulting in more intense meat color due to increased myoglobin content and elevated oxygen consumption (Jeong et al., 2009; Tian et al., 2021). Furthermore, the supplementation of the antibiotic tended to decrease carcass drip loss, which is commercially valuable because lower drip loss is associated with higher water-holding capacity, improving meat’s appearance, processing quality, and juiciness (Otto et al., 2004). Generally, oxidative damage can reduce water holding capacity and increase drip loss of meat, but antioxidant capacity of meat might have been improved by the supplementation of antibiotics (Traore et al., 2012; Ma et al., 2019). However, the effects of the antibiotic on meat color and carcass drip loss observed in this study were inconsistent with previous studies, which reported no significant effect (Ma et al., 2021; Tian et al., 2021; Ludwiczak et al., 2024). Additionally, the impact of antibiotics on meat quality in pigs remains difficult to interpret because their primary site of action is the intestinal tract and their impact on meat quality is likely to be indirect.

## Conclusions

Feeding growing pigs a diet supplemented with the antibiotic increased the weight gain resulting in increased carcass weight, whereas supplementing the herb and essential oil-based phytobiotic blend improved feed efficiency without affecting the weight gain. Increased feed efficiency may be partly attributed to the tendency reduction of jejunal TNF-α, a pro-inflammatory cytokine, possibly allowing reduced nutrient costs for immune reaction and efficient nutrient utilization even though it should be noted that TNF-α was the only cytokine measured limiting the scope of interpretation. Overall, this study suggests that the herb and essential oil-based phytobiotic may be a potential alternative to the antibiotic, but the specific mechanisms of the antibiotic and HEP may differ.

## Abbreviations:

HEP: herb and essential oil-based phytobiotic blend
TNF-α: tumor necrosis factor-α
